# Molecular characteristics of Polish field strains of Marek's disease herpesvirus isolated from vaccinated chickens

**DOI:** 10.1186/1751-0147-53-10

**Published:** 2011-02-14

**Authors:** Grzegorz Woźniakowski, Elżbieta Samorek-Salamonowicz, Wojciech Kozdruń

**Affiliations:** 1 National Veterinary Research Institute, Department of Poultry Viral Diseases, Partyzantow 57 Avenue, 24-100 Pulawy, Poland

## Abstract

**Background:**

Twenty-nine Marek's disease virus (MDV) strains were isolated during a 3 year period (2007-2010) from vaccinated and infected chicken flocks in Poland. These strains had caused severe clinical symptoms and lesions. In spite of proper vaccination with mono- or bivalent vaccines against Marek's disease (MD), the chickens developed symptoms of MD with paralysis.

Because of this we decided to investigate possible changes and mutations in the field strains that could potentially increase their virulence. We supposed that such mutations may have been caused by recombination with retroviruses of poultry - especially reticuloendotheliosis virus (REV).

**Methods:**

In order to detect the possible reasons of recent changes in virulence of MDV strains, polymerase chain reaction (PCR) analyses for *meq *oncogene and for long-terminal repeat (LTR) region of REV were conducted. The obtained PCR products were sequenced and compared with other MDV and REV strains isolated worldwide and accessible in the GeneBank database.

**Results:**

Sequencing of the *meq *oncogene showed a 68 basepair insertion and frame shift within 12 of 24 field strains. Interestingly, the analyses also showed 0.78, 0.8, 0.82, 1.6 kb and other random LTR-REV insertions into the MDV genome in 28 of 29 of strains. These genetic inserts were present after passage in chicken embryo kidney cells suggesting LTR integration into a non-functional region of the MDV genome.

**Conclusion:**

The results indicate the presence of a recombination between MDV and REV under field conditions in Polish chicken farms. The genetic changes within the MDV genome may influence the virus replication and its features *in vivo*. However, there is no evidence that *meq *alteration and REV insertions are related to the strains' virulence.

## Background

Neoplastic diseases of poultry may be related to infection with Marek's disease virus (MDV) and with the group of avian retroviruses. MDV is a highly cell-associated herpesvirus with lymphotropic properties but its molecular and genomic structures are similar to *Alphaherpesvirinae *subfamily [1].

The MDV's genome consists of double stranded DNA about 180 kb and it is divided into unique long (U_L_) and unique short (U_s_) regions flanked by internal (ITR) and terminal (TR) repeats.

The main oncoprotein of MDV is MEQ with leucine zipper characteristic for all transcriptional regulators and potential oncogens. MEQ is present in two copies located within the TR_L _region and between the junction of IR_L _and IR_s_. Other MDV genes taking part in replication and oncogenesis are ICP4 (immediately-early promoter), pp38 (phosphoprotein from pp38-pp24 complex) and LAT (latency associated transcripts), which down regulate MDV replication during latency [2]. The MDV genome is known to be susceptible to recombination and insertion of parts of foreign viral genomes especially retroviruses like the avian leukosis virus (ALV) and reticuloendotheliosis virus (REV) [3-5].

Retroviruses are another group of oncogenic viruses. The *Retroviridae *family falls into a few genera. ALVs are classified into *Alpharetrovirus *[6] whereas the REV group is in the separate *Gammaretrovirus *genus. However the structure and organization of their genome are common. The genomes of retroviruses consist of single-stranded RNA with positive polarity and present in two copies.

REV is an immunosuppressive virus affecting both humoral and cellular immunity [7]. An additional problem with REV is their ability to integrate a part or entire genome into the DNA of MDV [3,8]. This phenomenon occurs under field conditions [9]. The influence of a LTR-REV insertion into the MDV genome may change viral oncogenity or attenuation.

The main objective of this study was to determine molecular characteristics of recently isolated Polish strains of MDV and to identify factors responsible for changes of MDV virulence; changes that are commonly associated with mutation of the nucleotide sequence of *meq *oncogene [10].

## Methods

### Viruses

A total of 29 MDV field strains isolated from spleens of spontaneously infected vaccinated chickens, the HPRS_16 _reference strain (Houghton Poultry Research Station, Huntingdon. UK), the CVI988 Rispens strain from a commercial vaccine (Mérial, Lyon, France) and two BAC clones of Polish strains were propagated in chicken embryo kidney cells (CEKs) prepared from SPF chicken embryos (LTZ, Cuxhaven, Germany). A REV-T strain (National Veterinary Research Institute, Pulawy, Poland) was included in the study. The growth medium MEM (Gibco, Paisley, Scotland) supplemented with 10% bovine foetal serum and 1% antibiotics (Antibiotic-antimycotic, Gibco) was used, while a maintenance medium consisting of MEM with 1% mixture of antibiotics as above was used. MDV strains were incubated at 37.8°C in 5% CO_2 _until a cytophatic effect (CPE) was observed. The REV-T strain was also cultivated in CEKs and harvested 5 days post inoculation Third passage of each strain in CEKs was suspended in preservative medium containing 10% DMSO, gradually chilled and finally frozen in liquid nitrogen. The strains are listed in Table 1. The purity of the MDV strains from REV contamination was tested by filtering supernatants from the 3^rd ^passage of each MDV strain through the 0.2 μM syringe filters (Sartorious, Goettingen, Germany) and inoculating monolayers of CEKs. These cell cultures were incubated for 14 days in conditions as described above. Additionally DNA extracted from supernatants was tested with real-time polymerase chain reaction (real-time PCR) for the *gag *gene of REV according to the procedure developed by Hauck *et al. *[11] for excluding incidental contamination with REV.

**Table 1:** Marek's disease virus strains used in the study.

Number	Name of strain	Origin of strain
**1**	23_07_PL	16-w layers, spleen

**2**	25_07_PL	20-w breeders, spleen

**3**	29_07_PL	16-w layers, spleen

**4**	30_07_PL	17-w breeders, spleen

**5**	31_07_PL	27-w layers, spleen

**6**	32_07_PL	27-w layers, spleen

**7**	2_08_PL	28-w layers, spleen

**8**	3_08_PL	20-w layers, spleen

**9**	7_08_PL	26-w layers, spleen

**10**	8_08_PL	29-w layers, spleen

**11**	45_08_PL	19-w breeders, spleen

**12**	48_08_PL	15-w breeders, spleen

**13**	56_08_PL	21-w layers, spleen

**14**	73_08_PL	6-w broilers, spleen

**15**	8_09_PL	5-w broilers, spleen

**16**	23_09_PL	17-w layers, spleen

**17**	26_09_PL	32-w layers, spleen

**18**	40_09_PL	18-w breeders, spleen

**19**	42_09_PL	7-w broilers, spleen

**20**	45_09_PL	18-w layers, spleen

**21**	51_09_PL	18-w layers, spleen

**22**	81_09_PL	26-w layers, spleen

**23**	82_09_PL	25-w layers, spleen

**24**	86_09_PL	28-w layers, spleen

**25**	8_10_PL	24-w layers, spleen

**26**	10_10_PL	19-w layers, spleen

**27**	11_10_PL	25-w layers, spleen

**28**	30_10_PL	27-w layers, spleen

**29**	61_10_PL	6-w broilers, spleen

**30**	BAC_31_07Δ*meq*	BAC-clone from *Escherichia coli *GST1763 cells

**31**	BAC_7_08	BAC clone from *Escherichia coli *GST1763 cells

**32**	HPRS_**16**_	Houghton Poultry Research Station, Huntingdon UK

**33**	CVI/988 Rispens	Commercial vaccine (Mérial)

All strains were passed 3 times in chicken embryo kidney cells.

W: week-old, CEK: chicken embryo kidney cells, BAC_31_07Δ*meq*: deletive strain of 31_07 strain in bacterial artificial chromosome.

### DNA extraction

Total DNA was extracted from infected CEKs using QIAamp Mini Kit (Qiagen, Hilden, Germany) according to the manufacturer's procedure.

### Real-time PCR

Primers complementary to ICP4, pp38 and *meq *oncogene were used [2]. For the normalisation of results as well as for calculation of viral copy number per 10^4 ^infected cells, the chicken ovotransferrin gene was also amplified for each sample. Each sample was run in duplicate. Reaction mixture, standards and conditions of real-time PCR has been described elsewhere [2]. As positive controls, HPRS_16 _and Rispens strains as well as MDV BAC-clones were used.

### PCR

PCR with primers complementary to *meq *was applied as previously described [10]. The PCR products were purified using Nucleospin Kit (Macherey-Nagel, Düren, Germany).

### PCR for LTR

The sequences of primers specific for LTR-REV were: LTR U3 REV: 5'-CCG AGA AAT GAT ATC AGC-3' and U5 REV: 5-GGT GGG GTA GGG ATC CG-3' [9]. The reaction volume was 25 μL and contained: 2.5 μL of 10-fold concentrated PCR buffer (1.5 mM MgCl_2_), 1 μL of dNTPs (0.2 μM of each dNTP), 0.5 μL HotStar *Taq *Polymerase (2.5 U) (Qiagen), 5 μL of 1M betaine solution (0.2 μM) (Sigma-Aldrich, Munich, Germany), 1 μL of each primer (0.4 μM), 1 μL of total DNA and 13 μL of deionised water. PCR conditions were as following: initial denaturation - 95°C for 5 min., following 35 cycles of: exact denaturation 94°C for 1 min., primers annealing 62°C for 1 min., products extension 72°C - 1 min., final extension of the products 72°C for 10 min. Positive control for PCR was extracted from CEKs inoculated with the REV-T strain while a negative control was extracted from non-inoculated cell cultures.

### DNA sequencing

PCR products of *meq *from 24 MDV strains and 10 products for the LTR region were sequenced from forward and reversed primers on GS FLX/Titanium sequencer (Roche, Branford, Connecticut, USA) by GENOMED (Warsaw, Poland). Each amplicon was run 2 times to ensure the reliability of the results. The *meq *and LTR sequences were assembled into single contig in Bioedit ver. 7.0.9.0 then submitted to NCBI Genebank and aligned using Geneious ver. 5.1 [12]. PCR products were separated in 1.5% ethidium bromide stained agarose gels.

## Results

As the first step of this study, the examined MDV field strains were propagated in CEKs. Three passages of these strains were conducted. CPE was observed in all infected cell cultures starting from the 96^th ^h of 3^rd ^passage. An early CPE was seen as small round cells reflecting light waves. These cells formed foci and syncytias that detached from the wall of cell culture flask causing formation of plaques. From these passaged MDV strains, total DNA was extracted. Similarly, DNA extracted from CEKs infected with REV-T was used as a positive control. There was no CPE observed in CEKs inoculated with filtered supernatants taken from cell cultures infected with MDV thus confirming the purity of the used strains. Similarly, the results of real-time PCR for the detection of *gag *gene of REV in supernatants were negative.

Next, real-time PCR was conducted on all DNA samples extracted from infected CEKs. The specific fluorescent signal observed as curves for ICP4, pp38 and *meq *genes were detected in all samples. On the basis of quantification of ICP4, pp38 and *meq*, the number of viral copies was determined as ranging from 63 ± 2 to 12123 ± 587 copies per 10^4 ^infected cells.

### *Meq *analysis

**Figure 1:**
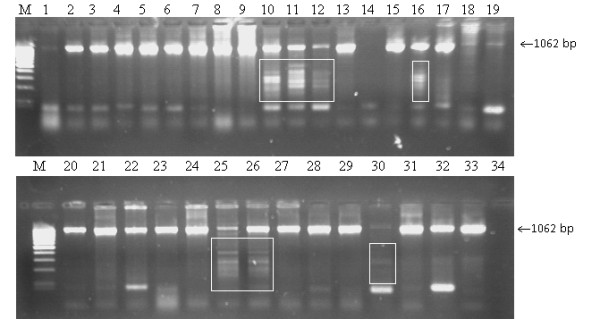
**PCR results for the detection of the *meq *oncogene in Marek's disease virus**. M-Mass Ruller 100 kb Plus (80 bp-10 000 bp), Fermentas, 1 - 23_07_PL, 2 - 25_07_PL, 3 - 29_07_PL, 4 - 30_07_PL, 5 - 31_07_PL, 6 - 32_07_PL, 7 - 2_08_PL, 8 - 3_08_PL, 9 - 7_08_PL, 10 - 8_08_PL, 11 - 45_08_PL, 12 - 48_08_PL, 13 - 56_08_PL, 14 - 73_08_PL, 15 - 8_09_PL, 16 - 23_09_PL, 17 - 26_09_PL, 18 - 40_09_PL, 19 - 42_09_PL, 20 - 45_09_PL, 21 - 51_09_PL, 22 - 81_09_PL, 23 - 82_09_PL, 24 - 86_09_PL, 25 - 8_10_PL, 26 - 10_10_PL, 27 - 11_10_PL, 28 - 30_10_PL, 29 - 61_10_PL, 30 - BAC_31_07Δ*meq*, 31 - BAC_7_08, 32 - HPRS_16_, 33 - Rispens, 34 - negative DNA control extracted from non-infected chicken embryo kidney cells. Smeared products are marked with a white box.

By standard PCR, the presence of an approximately 1062 bp long product for the *meq *oncogene was found in samples except sample 73_08 (Figure 1, lane 14) and BAC_31_07Δ*meq *(Figure 1, lane 30). In 5 samples (Figure 1, lanes: 1, 18, 19, 25 and 30) the PCR products were weak and for 7 samples, additional smears in the background were observed (Figure 1, lanes: 10, 11, 12, 16, 25, 26, 30) as fine bands ranging from 210 to 550 bp. PCR products for *meq *oncogene were sequenced and submitted to NCBI Genebank (Table 2).

**Table 2:** Sequences of *meq *oncogene and long terminal repeats sequence fragment of reticuloendotheliosis virus genome submitted to NCBI Genebank database.

Strain	Accession number	Strain	Accession number
25_07_PL	Genebank:HQ204792	3_08_PL	Genebank:HQ204809

8_10_PL	Genebank:HQ204793	7_08_PL	Genebank:HQ204810

10_10_PL	Genebank:HQ204794	8_08_PL	Genebank:HQ204811

11_10_PL	Genebank:HQ204795	12_08_PL	Genebank:HQ204812

30_10_PL	Genebank:HQ204796	45_08_PL	Genebank:HQ204813

8_09_PL	Genebank:HQ204797	56_08_PL	Genebank:HQ204814

23_09_PL	Genebank:HQ204798	73_08_PL	Genebank:HQ204815

40_09_PL	Genebank:HQ204799	LTR 7_08	Genebank:HQ204816

42_09_PL	Genebank:HQ204800	LTR 11_10	Genebank:HQ204817

45_09_PL	Genebank:HQ204801	LTR 29_07	Genebank:HQ204818

51_09_PL	Genebank:HQ204802	LTR 31_07	Genebank:HQ204819

81_09_PL	Genebank:HQ204803	LTR 30_10	Genebank:HQ204820

82_09_PL	Genebank:HQ204804	LTR 42_09	Genebank:HQ204821

29_07_PL	Genebank:HQ204805	LTR 45_08	Genebank:HQ204822

31_07_PL	Genebank:HQ204806	LTR 45_09	Genebank:HQ204823

32_07_PL	Genebank:HQ204807	LTR 51_09	Genebank:HQ204824

2_08_PL	Genebank:HQ204808	LTR 82_09	Genebank:HQ204825

**Figure 2:**
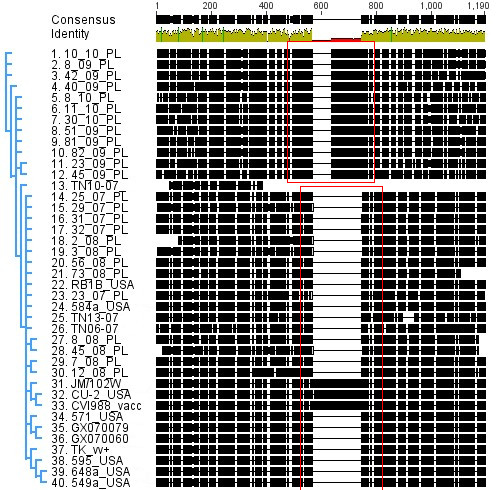
**Nucleotide alignment for *meq *oncogene of Polish Marek's disease virus (MDV) field strains and reference strains**. The difference between strains isolated between 2007 and 2009 and 12 of 24 MDV strains (isolated 2009-2010) are marked with a red box (Geneious ver 5.1).

Analysis of *meq *showed a 68 bp insertion in position 566 of *meq *in 12 of 24 strains (Figure 2). These insertions resulted in a frame shift in Polish field strains isolated in 2009 and 2010. Other minor single nucleotide substitutions were also detected in 12 strains without influencing the amino-acid composition of MEQ. Nucleotide alignment with *meq *sequences accessible in Genebank was performed. DNA sequences of *meq *shared 78.9% homolology of gene sites.

### LTR analysis

**Figure 3:**
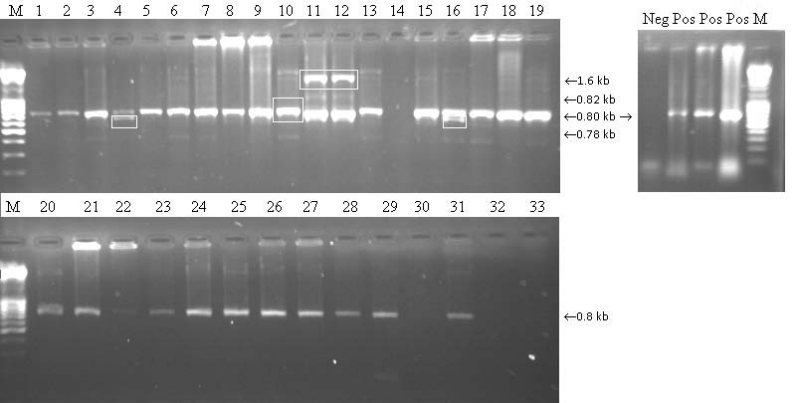
**PCR for the detection of reticuloendotheliosis virus (REV) long terminal repeats**. M-Mass Ruller 100 kb Plus (80 base pairs - 10,000 base pairs), Fermentas, Neg- negative DNA control extracted from non-infected chicken embryo kidney cells, Pos - replicates of DNA control extracted from the infected chicken embryo kidney cell cultures with REV-T strain 5 days post inoculation. All three positives are derived from the same original strain. 1 - 23_07_PL, 2 - 25_07_PL, 3 - 29_07_PL, 4 - 30_07_PL, 5 - 31_07_PL, 6 - 32_07_PL, 7 - 2_08_PL, 8 - 3_08_PL, 9 - 7_08_PL, 10 - 8_08_PL, 11 - 45_08_PL, 12 - 48_08_PL, 13 - 56_08_PL, 14 - 73_08_PL, 15 - 8_09_PL, 16 - 23_09_PL, 17 - 26_09_PL, 18 - 40_09_PL, 19 - 42_09_PL, 20 - 45_09_PL, 21 - 51_09_PL, 22 - 81_09_PL, 23 - 82_09_PL, 24 - 86_09_PL, 25 - 8_10_PL, 26 - 10_10_PL, 27 - 11_10_PL, 28 - 30_10_PL, 29 - 61_10_PL, 30 - BAC_31_07Δ*meq*, 31 - BAC_7_08, 32 - HPRS_16_, 33 - Rispens, random insertions into Marek's disease virus genomes are marked with white boxes.

PCR for LTR of REV was specific for DNA extracted from CEKs infected with the REV-T strain and showed the presence of an about 0.8 kb insertion in 28 MDV strains (Figure 3). The fragment was not observed in two strains (Figure 3, lanes 14 and 30). An additional insertion of LTR-REV about 0.78 kb into the MDV genome was detected in two strains (Figure 3, lanes 4 and 16). Two other strains showed the presence of an additional 1.6 kb PCR product (Figure 3, lanes 11 and 12). Moreover in two strains (Figure 3, lanes 10 and 13) the LTR insertion was 20 bp longer than in other strains harboring the 0.8 bp fragment. Except for the 0.8 kb product in DNA of the 21 strains, a fine PCR ladder of PCR products ranging from about 0.8 kb to 2.0 kb was observed in the background.

LTR fragments were sequenced and submitted to NCBI Genebank. The accession numbers are given in Table 2. The sequences were aligned with other REV sequences accessible in NCBI Genebank.

**Figure 4:**
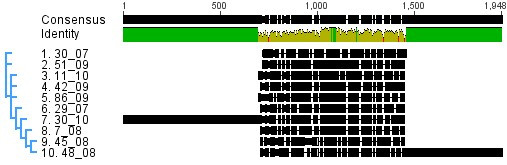
**Nucleotide alignment of long terminal repeat inserts from 10 Polish Marek's disease virus field strains (Geneious ver 5.1)**.

Sequencing of 10 out of 28 MDV recombinant samples confirmed the finding that MDV strains occurring in Poland have a LTR-REV insertion. These sequences shared 70.1% of LTR-REV sequence homology. An alignment of MDV-LTR fragments is shown on Figure 4. Inserts of two chosen strains were significantly longer than in case of the other 8 strains.

## Discussion

Our previous study on the molecular characteristics of Polish MDV strains isolated during 2007-2009 demonstrated a close correlation between changes in nucleotide and amino-acid sequences and the strain virulence [10]. The present study expands the knowledge on the molecular characteristics of Polish MDV strains. The obtained results from sequencing of genes in 24 strains suggested a possible frame shift being detected in 12 of the recently isolated field strains. Interestingly, these strains were mainly found in chickens from the south-western parts of Poland, where cases of chicken lymphomas have been observed in the past [personal observation E Samorek-Salamonowicz]. Strains not having a frame shift in their genome originated from the western regions of Poland close to the German border. The incidence of REV infections in Poland is unknown. However it is assumed, e.g. based on veterinary reports that the incidence is higher in southern Poland where poultry farming is most intensive. It is known that MDV may co-exist with other retroviruses, especially ALV and REV in chicken cells [1,5,6]. *In vitro *recombination of these viruses and MDV in chicken and duck embryo fibroblasts has been reported [4,8]. The 3 passages of the strains isolated from chickens with clinical MD revealed a persistent presence of LTR in the MDV genomes. A stable integration between MDV and REV is possible as previously discussed by Jones *et al*. [4]. *In vivo *studies on the molecular and biological features of MDV-REV BAC clone GX0101 and its virulence for chickens were studied by Sun *et al*. [13,14]. However the consequences of a LTR insertion into the IRs and Us junction of MDV still remains unknown. The presented study of Polish MDV strains was done using heterogeneous isolates. The obtained two BACs constructed from these strains will be a good model for further study of the effects of these insertions. In spite of detection of parallel changes in *meq *and LTR insertions into the MDV genome, their role remains unexplained. However, LTR insertions may affect genes of importance for MDV infection mechanisms. We will investigate this in future studies using BACs. The insertions present in 12 of the 24 Polish field strains suggest that this is a common event in the high density poultry industry. In this study real-time PCR for the detection of the MDV specific amplicons like ICP4, pp38 and a part of *meq *revealed no influence of LTR insertion on the molecular features of these genes. However LTR insertions were also present 100-200 bp downstream from the *ICP4 *gene [15]. Therefore a LTR insertion may alter also basis transactivation function by *ICP4*. Moreover, the full sequencing of *meq *oncogene conducted in our study and the analysis of the assembled sequences showed a 68 bp mutation within this gene. The insertion was located at the nucleotide 565 of *meq*. Similarly, in the same position a longer 180 bp insertion was observed in attenuated strains (CU-2, JM/102W and CVI988 Rispens). Interestingly, MDV strains with the 68 bp insertion were isolated in the south-western provinces of Poland where retroviral infections in poultry have been recognized previously. It is however possible that the phenomena occurred independently. But it might also suggest an association of the insertion with changes in virulence. The virulence of the isolated MDV strains were not determined in this study but will be examined in the future. However a rather high level of virulence is expected as the strains were isolated from chickens with severe clinical symptoms and MD lesions.

The 0.8 kb LTR insertion into the MDV genome was detected in 28 strains while 2 strains also had an insertion being visible as a 1.6 kb band. This insertion could be assigned as a most efficiently transcribed LTR region within *SORF2 *region of MDV [4]. *SORF2 *is a homologue of *US22 *gene of human cytomegalovirus and human herpesvirus 6 EPLF3 [4,16]. This insertion may alter transactivation of MDV genes as suggested in previous studies [8,9]. The multiple bands observed in a few Polish field strains suggest different sites of LTR integration in the U_S _MDV region. Jones *et al*. [8] described smeared bands as randomly integrated REV proviruses. This may influence the sequence and structure of different MDV genes including *meq*. However the smears in the background of the deletive BAC_31_07Δ*meq *suggest that the insertions are not necessarily associated only with *meq *(Figure 1). The sequenced LTR fragments of 10 strains were new in NCBI Genebank. The changes detected within *meq *are confirmed by the finding that LTR insertions are often located near to proto-oncogenes thus being able to activate tumoral processes [3]. An other scenario is that IR_S _and U_s _insertions may alter the recombination of chimeric viruses *in vitro *and *in vivo *since the LTR-REV regions are known as strong enhancers of promoters in chicken embryo fibroblasts [17]. However confirmation of these hypotheses needs further studies. Previous reports on the effects of a LTR-REV insertion into RM1 [1] or GX0101 [13,14] strains of MDV suggest attenuation and reduction of their oncogenicity with intact replication abilities. However insertions in Polish MDV strains seemed not to influence their oncogenicity since they caused outbreaks of MD in vaccinated chickens. The exact effects of these insertions will be studied experimentally after establishing BAC clones lacking the LTR fragment. The LTR insertions in MDV could make the epidemiology of Marek's disease more complex. These aspects will be investigated in future studies.

## Conclusions

Significant insertions within *meq *oncogene sequence were detected among recently isolated virulent MDV strains from Poland. The detected insertional mutation may lead to structural and functional changes of multiple proteins and virus features.

## List of abbreviations

ALV: Avian leukosis virus; BAC: bacterial artificial chromosome including MDV genome; bp: base pair; CEK: chicken embryo kidney cells; DMSO: dimethyl sulfoxide; EPLF3: gene of human herpesvirus 6; IR_L_: internal repeated long sequence of MDV; IR_s_: internal repeated short sequence of MDV; kbp: thousand of base pairs; LTR: long terminal repeats of REV genome; LTZ: Lohmann Tierzucht Company, Germany; MEM: minimal essential medium; MEQ: main oncoprotein of MDV crucial in tumorigenesis; MDV: Marek's disease virus; REV: reticuloendotheliosis virus; SPF: specific pathogen free chickens. The following pathogens are certified to be absent: avian adenoviruses, infectious bronchitis virus, infectious laryngotracheitis virus, avianleukosis, avian reticuloendotheliosis and Marek's disease virus; SORF2: short opened reading frame 2 of MDV; TR_s_: terminal repeated short sequence of MDV; TR_L_: terminal repeated long sequence of MDV; U_s_: unique short region of MDV; U_L_: unique long region of MDV; US22: gene located within unique short region of MDV; ver. -version.

## Competing interests

The authors declare that they have no competing interests.

## Authors' contributions

GW carried out isolation of the used strains, DNA extraction, PCRs, sequencing of *meq *and LTR genes and nucleotide alignments. ESS and WK participated in DNA extraction, real-time PCRs, PCRs and assesment of the final results. ESS provided personal observations used in the present study. WK provided detailed information regarding origin of used MDV field strains. ESS and WK participated in coordination of the study. The final manuscript was read and approved by all authors.
